# Sexually Dimorphic Risk Mitigation Strategies in Rats

**DOI:** 10.1523/ENEURO.0288-16.2017

**Published:** 2017-02-06

**Authors:** Blake A. Pellman, Bryan P. Schuessler, Mohini Tellakat, Jeansok J. Kim

**Affiliations:** 1 Department of Psychology, University of Washington, Seattle, Washington 98195-1525; 2 Department of Psychology, University of Texas at Austin, Austin, Texas 78712; 3 Program in Neuroscience, University of Washington, Seattle, Washington 98195-1525

**Keywords:** anxiety, decision-making, fear, foraging behavior, sex differences

## Abstract

The scientific understanding of fear and anxiety—in both normal and pathological forms—is presently limited by a predominance of studies that use male animals and Pavlovian fear conditioning-centered paradigms that restrict and assess specific behaviors (e.g., freezing) over brief sampling periods and overlook the broader contributions of the spatiotemporal context to an animal’s behavioral responses to threats. Here, we use a risky “closed economy” system, in which the need to acquire food and water and the need to avoid threats are simultaneously integrated into the lives of rats, to examine sex differences in mitigating threat risk while foraging. Rats lived for an extended period (∼2 months) in enlarged chambers that consisted of a safe, bedded nest and a risky foraging area where footshocks could be delivered unpredictably. We observed that male and female rats used different strategies for responding to the threat of footshock. Whereas male rats increased the size of meals consumed to reduce the overall time spent foraging, female rats sacrificed their metabolic needs in order to avoid shocks. Ovarian hormone fluctuations were shown to exert slight but reliable rhythmic effects on risky decision-making in gonadally intact female rats. However, this did not produce significant differences in approach–avoidance trade-offs between ovariectomized and intact female groups, suggesting that male–female sex differences are not due to the activational effects of gonadal hormones but, rather, are likely to be organized during early development.

## Significance Statement

The National Institutes of Health (NIH) has recently mandated that all NIH-funded research must include balanced samples of each sex, but the initiative does little to address the reasons why researchers have avoided including female animals in their studies, which includes the perceived difficulty and high cost of controlling for estrous cycle-related variability and its effect on behavior. Here, we use a longitudinal design to measure sex differences and the temporal variability of ovarian hormones on fear, anxiety, and risky decision-making continuously, and demonstrate functional differences in fear and anxiety behaviors between male and female rats that are independent of estrous cycle fluctuations.

## Introduction

Anxiety- and fear-related disorders are among the most common mental illnesses, usually afflicting women significantly more than men (Kessler et al., 2005), and the United States spends millions of dollars each year for researching the basic mechanisms of normal and pathological fear and anxiety (Institute of Medicine, 2014). Typical animal models of anxiety- and fear-related disorders predominately use behavioral paradigms, such as Pavlovian fear conditioning, and open field and elevated plus maze tests, which are designed to assess a specific behavior (e.g., freezing) during brief sampling periods (Davis, 1992; Lissek et al., 2005; Milad and Quirk, 2012; Jones and Monfils, 2016). While these paradigms to study fear and anxiety have been useful in generating much of the knowledge we have on the mechanisms and influence of various treatments (e.g., drugs, stress, social interactions), all such studies are similarly limited in that they examine behavior in settings that restrict the natural repertoire of behaviors (largely to simplify analysis) and sample behavior over a short duration, which ignores the longer-scale spatiotemporal integration of information that animals rely on to function in natural environments.

Compounding these limitations, there is a lack of representation of female animals in fear and anxiety research, caused in part by the perceived difficulty and cost in controlling the variability associated with female animals’ reproductive cycles (Zucker and Beery, 2010; Prendergast et al., 2014; Becker et al., 2016). The estrous cycle of a female rat, which is typically divided into four primary “stages” (proestrus, estrus, metestrus, and diestrus), has been linked to physiological fluctuations in synapse density in the hippocampus, amygdala, and prefrontal cortex (Woolley and McEwen, 1992; Shansky et al., 2004; Rasia-Filho et al., 2012), neurogenesis in the hippocampus (Pawluski et al., 2009), and behavioral variation in elevated plus-maze (Marcondes et al., 2001), open field (Frye and Walf, 2002), fear conditioning (Markus and Zecevic, 1997; Gupta et al., 2001), and fear extinction tests (Milad et al., 2009). Completely controlling for such fluctuations systematically would require testing animals in all test stage–estrous phase combinations, demanding greater animal numbers and substantially increasing costs. Nonetheless, brief temporal sampling and the lack of female representation have likely led to a narrow view of fear and anxiety processes, and the extent to which animal models of these processes generalize to those of humans is questionable. Indeed, studies have found that male rats show greater fear responses than female rats (Maren et al., 1994; Markus and Zecevic, 1997), which does not parallel either the findings that women have higher incidences of and suffer more from anxiety- and fear-related disorders than men (Brown et al., 1992; McLean and Anderson, 2009), or that, even under normal conditions, women report experiencing stronger fears than men (Geer, 1965; Ollendick et al., 1989; Gullone, 2000).

**Figure 1.**
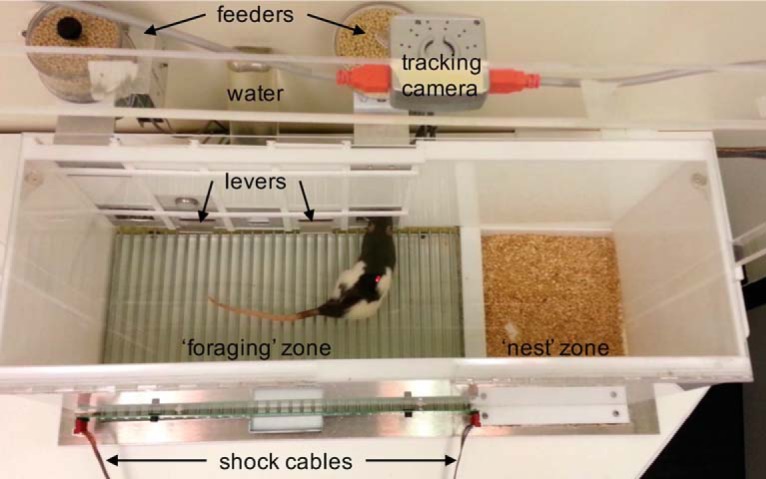
Experimental apparatus and design of the closed economy. Photograph of a closed economy box. Measurements of lever presses, food dispensed, water bottle licks, footshocks delivered and received, and locomotor tracking and spatial position via overhead-mounted tracking camera were coordinated by ANY-maze I/O interface and software (RRID:SCR_014289). Gonadally intact male (*n* = 8) and female (*n* = 12) and OVX female rats (*n* = 8) rats were housed in the closed economy boxes upon arrival and were shaped to respond on an FR25-CRF schedule over ∼12 d, followed by 14 d of baseline measurement, 14 d of shock, and 14 d of extinction (no shock) conditions.

The present study used a longitudinal, risky “closed economy” system (Hursh, 1980; Fanselow and Lester, 1988; Helmstetter and Fanselow, 1993; Kim et al., 2014; Pellman et al., 2015) to investigate sex differences and the activational role of ovarian hormones in fear and anxiety behaviors in rats. The risky closed economy system attempts to model an approach–avoidance conflict ostensibly shared by all species: decisions to explore and forage for resources, such as food and water, must be balanced by decisions to avoid potential risks and the threat of predation (Lima and Dill, 1990). This was modeled by housing rats in a dynamic environment that consisted of a safe, bedded nest area and a risky foraging area that had to be entered in order to obtain food and water and where footshocks could be delivered unpredictably (Fig. 1). It was hypothesized that female rats will be more reactive to the shock condition, in terms of foraging and avoidance behavior, compared with male rats. Furthermore, female rats will exhibit rhythmic variation in their risk-taking behaviors related to the fluctuations of ovarian hormones that will not be observed in male or ovariectomized female rats.

## Materials and Methods

### Subjects

Experimentally naive, gonadally intact male (*n* = 8) and female (*n* = 12) and ovariectomized (OVX) female (*n* = 8) Long–Evans rats (catalog #2308852, RGD; RRID:RGD_2308852) initially 7–8 weeks of age were individually housed in eight closed economy chambers (Fig. 1) distributed between two separate rooms within the Department of Psychology at the University of Washington (accredited by the Association for Assessment and Accreditation of Laboratory Animal Care). Animals were tested in cohorts of eight, with experimental groups counterbalanced between the two rooms, and male and female rats within each cohort were kept in separate rooms. Ovariectomy surgeries were performed by Charles River Laboratories (RRID:SCR_003792) the day prior to shipment. All animal experiments were conducted in compliance with the National Institutes of Health *Guide for the Care and Use of Laboratory Animals* and were reviewed and approved by the University of Washington Institutional Animal Care and Use Committee.

### Closed economy task

The closed economy dimensions were 74.3 × 25.4 × 33 cm (length × width × height) and consisted of a “nest” (20.3 × 25.4 cm) and a “foraging arena” (54 × 25.4 cm). The nest floor was covered with sawdust, while the floor of the foraging arena was composed of 32 stainless steel rods (4.5 mm diameter) wired to a precision animal shocker (Coulbourn Instruments) for delivery of footshocks. A camera (Fire-I B/W Board camera; Unibrain) was mounted above each closed economy chamber and connected to a computer for tracking animal activity via ANY-maze software (RRID:SCR_014289), which also measured the activation of the food levers and dispensers (Med Associates) and the shock generator connected to an ANY-maze Interface (AMi; Stoelting). Forty-five milligram grain-based pellets for rodents (catalog #F0165, Bio-Serv) were used for food. White noise (70 dB) generated by the ANY-maze software was continuously played through computer speakers throughout the experiment to obscure external noises.

After arrival, animals were given 12-14 d to acclimatize to the chambers and light cycle (12 h light/dark cycle), during which time they were shaped to press either of two levers 25 times initially to gain access to a continuous reinforcement schedule [fixed ratio 25-continuous reinforcement (FR25-CRF)], which was reset if 1 min had passed since the last lever press. The FR threshold was doubled every 2 d (except FR16 was increased to FR25) until stable lever pressing at the FR25 threshold was achieved. After 14 baseline days (“Baseline”), all of the animals were exposed to 14 d of unsignaled, pseudorandom footshocks (0.8 mA; ∼2 shocks/h; “Shock”). These shock parameters were chosen to maximize the changes in meal patterns and foraging strategies without creating an overly aversive environment that inhibits foraging altogether (Helmstetter and Fanselow, 1993). If the animal was in the foraging area when ANY-maze triggered the footshock, the shock continued for up to 10 s or until the animal escaped to the nest. If the animal was in the nest at the time the program generated the footshock, it terminated instantaneously. Animals were removed from the closed economy chambers and housed individually in a separate vivarium (also within the Department of Psychology at the University of Washington and accredited by the Association for Assessment and Accreditation of Laboratory Animal Care) during the last hour of the light phase [zeitgeber time 11 (ZT11) to ZT12] every 1–2 d so the chambers could be cleaned, the food and water replaced, and the animals weighed. They were provided access only to water in their temporary cages during this time.

### Estrous cycle monitoring

Estrous phase was determined daily via vaginal lavage (Marcondes et al., 2002) in a subset of female rats (*n* = 4). Rats were brought into a separate room individually and handled momentarily, then were held in place by hand, and a pipette filled with 10 µl of 0.9% saline solution was gently and shallowly inserted into the vagina. The saline was washed in and drawn out, and was immediately placed on slides for cytological examination under a light microscope. Furthermore, OVX female rats were confirmed to lack a functioning estrous cycle by monitoring vaginal cytology for a week at the end of the experiment, which was characterized by a lack of change in cytology across days (Monies and Luque, 1988).

### Statistical analyses

All data are presented as the mean ± SEM. The data from the closed economy experiment were analyzed using mixed-factorial ANOVAs on the daily means, except where noted, with the within-subjects factor of experimental condition (baseline, shock, or extinction) and the between-subjects factor of sex. In cases where the assumption of sphericity was violated (Mauchly’s test), Greenhouse–Geisser-corrected degrees of freedom were used. In cases where Levene’s test for equality of variance was significant, Kruskal–Wallis tests were used to test for group differences within each condition independently. Bonferroni’s test or Dunnett’s T3-adjusted (for samples with unequal variances), two-tailed, paired-samples *t* tests or independent *t* tests were used for *post hoc* tests where noted. To determine a discrete “darting episode,” ANY-maze was programmed to automatically detect movement speed that exceeded 23.5 cm/s, and movement speed had to decrease below 11.8 cm/s before another darting episode was counted. To analyze estrous cycle-related fluctuation of locomotor activity in the closed economy, the apparatus-wide activity of rats (distance traveled, in meters) was summed in 1 h time bins and broken down into its component frequencies using fast Fourier transforms (FFT). The magnitudes of the frequencies in the 3–5 d range were used to test for statistically significant sex differences using independent, one-tailed *t* tests. The appropriate group size (*n* = 4) for estrous cycle monitoring was determined by a power analysis (G*Power 3.1; RRID:SCR_013726), using an α of 0.05, power of 0.80, and an effect size (Cohen’s *f*) of 0.50, which was based on findings from previous studies examining the effect of estrous cycle on anxiety-related behaviors (Diaz-Veliz et al., 1989; Mora et al., 1996). Analyses were performed with SPSS 19 (RRID:SCR_002865), and graphs were generated with GraphPad Prism 7.0 (RRID:SCR_002798).

## Results

Gonadally intact, adult male (*n* = 8) and female (*n* = 12) rats and OVX adult female rats (*n* = 8) were housed individually in closed economy chambers (Fig. 1) upon arrival and maintained on a 12 h light/dark cycle. Subsequently, all animals were shaped to a FR25-CRF food schedule on either of two levers (i.e., after 25 lever presses, each lever press produced one pellet). These contingencies were reset if 1 min elapsed between any lever presses. Once stable responding had been reached, baseline measurements continued for 14 d (Baseline), followed by a 14 d period of pseudorandom, unpredictable footshocks delivered to the foraging area (∼2/h; Shock), and ending with a 14 d period where footshocks were terminated (“Extinction”).

**Figure 2.**
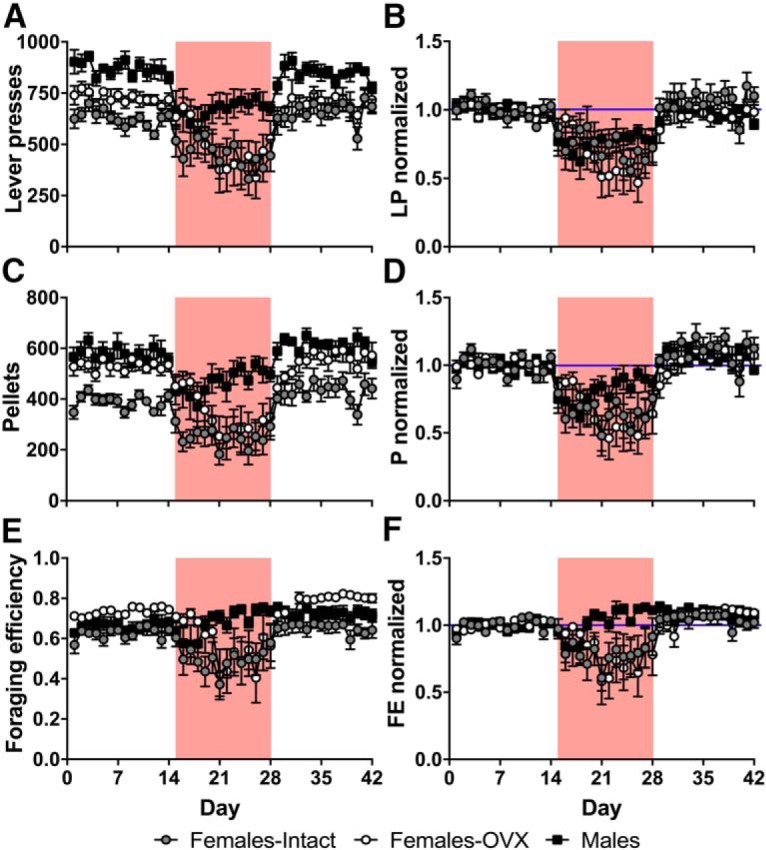
Sex differences and effects of ovariectomy and footshocks on foraging behaviors. ***A***, ***B***, Mean daily (***A***) and normalized (***B***; percentage of baseline average) number of lever presses (LPs) across both levers. ***C***, ***D***, mean daily (***C***) and normalized (***D***) number of pellets dispensed (P) across both dispensers. ***E***, ***F***, mean daily (***E***) and normalized (***F***) foraging efficiency (pellets/lever press; FE). Gray circles represent intact female rats (*n* = 8), open circles represent OVX female rats (*n* = 8), and black squares represent male rats (*n* = 8). Red background denotes shock period. All data represent the mean ± SEM.

**Figure 3.**
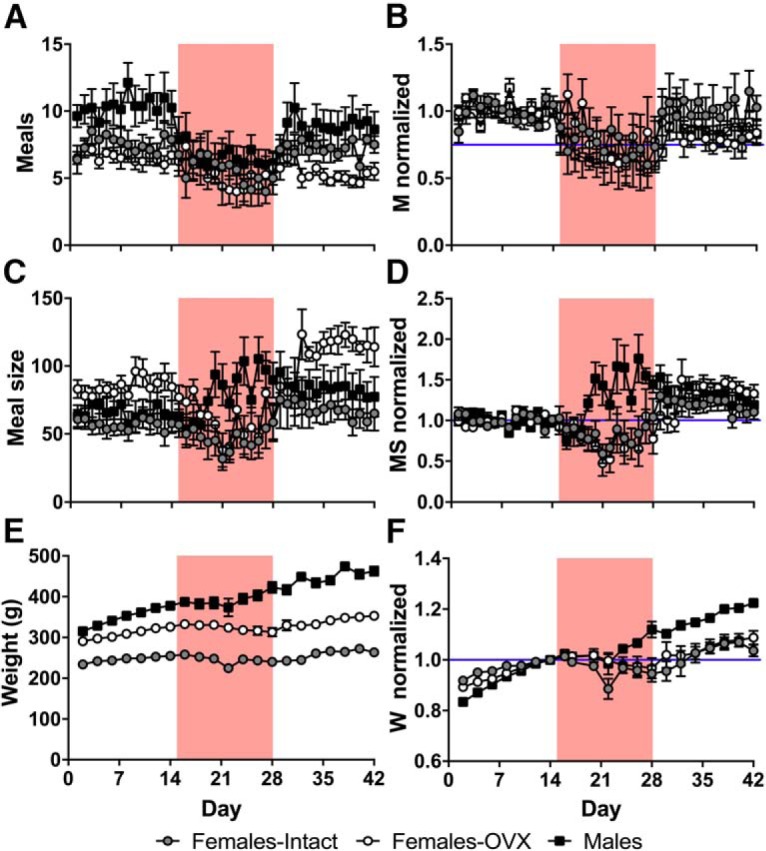
Sex differences and effects of ovariectomy and footshocks on meal patterns and body weight. ***A***, ***B***, Mean daily (***A***) and normalized (***B***; percentage of baseline average) number of discrete meals obtained (FR25 threshold reached; M) across both levers. ***C***, ***D***, mean daily (***C***) and normalized (***D***) meal sizes (pellets/meal; MS) across both levers. ***E***, ***F***, The 2 d mean (***E***) and normalized (***F***; percentage of last baseline 2 d mean) body weight (in grams; W). Gray circles represent intact female rats (*n* = 8), open circles represent OVX female rats (*n* = 8), and black squares represent male rats (*n* = 8). Red background denotes shock period. All data represent the mean ± SEM.

During baseline, male rats pressed levers at higher rates than both intact and OVX female rats, which did not differ from each other (Fig. 2*A*; group: *F*_(2,21)_ = 19.28, *p* = 0.000018; condition: *F*_(1.44,30.27)_ = 27.82, *p* = 0.000001, df adjusted; condition*group: *F*_(2.88,30.27)_ = 0.26, *p* = 0.85, df adjusted; *post hoc* tests: males vs intact: *p* = 0.000017; males vs OVX: *p* = 0.00088; intact vs OVX: *p* = 0.32, Bonferroni corrected). During the shock condition, although males continued lever pressing more than the females, all groups reduced lever pressing by a similar degree, and lever pressing quickly recovered to baseline levels during extinction (Fig. 2*B*; group: *F*_(2,21)_ = 0.68, *p* = 0.52; condition: *F*_(1.47,30.93)_ = 27.45, *p* = 0.000001, df adjusted; condition*group: *F*_(2.95,30.93)_ = 0.46, *p* = 0.71, df adjusted). Almost all animals exclusively preferred one of the two levers, and this preference did not change appreciably across conditions. During baseline, male rats obtained significantly more pellets than intact females, but not OVX females (Fig. 2*C*; group: *F*_(2,21)_ = 21.78, *p* = 0.000008; condition: *F*_(1.31,27.40)_ = 40.71, *p* = 1.28E-7, df adjusted; condition*group: *F*_(2.61,27.40)_ = 0.74, *p* = 0.52, df adjusted*; post hoc* tests: males vs intact: *p* = 0.00018; males vs OVX: *p* = 0.091; intact vs OVX: *p* = 0.0012, Dunnett T3 corrected), and there were no significant differences between groups in the change in the number of pellets acquired during shock and extinction conditions compared with baseline (Fig. 2*D*; group: *F*_(2,21)_ = 1.52, *p* = 0.24; condition: *F*_(1.29,26.98)_ = 41.31, *p* = 1.36E-7, df adjusted; condition*group: *F*_(2.57,26.98)_ = 1.09, *p* = 0.36, df adjusted). It is interesting to note that, although intact and OVX females pressed the levers at similar rates, OVX females were able to obtain significantly more food than intact females. Because obtaining pellets depends not just on lever pressing, but also on specifically uninterrupted lever pressing after reaching the FR25 threshold, a measure of “foraging efficiency” (pellets dispensed/lever presses) was created to estimate the proportion of lever presses that resulted in food pellets. Figure 2, *E* and *F*, indicates that OVX females were significantly more efficient at obtaining pellets than intact females during the baseline and extinction periods (group: *F*_(2,21)_ = 6.56, *p* = 0.0061; condition: *F*_(1.20,25.20)_ = 18.78, *p* = 0.00010, df adjusted; condition*group: *F*_(2.40,25.20)_ = 3.21, *p* = 0.050, df adjusted; *post hoc* tests: Baseline: males vs intact, *p* = 0.60; males vs OVX, *p* = 0.12; intact vs OVX, *p* = 0.0055; Shock: males vs intact, *p* = 0.036; males vs OVX, *p* = 0.45; intact vs OVX, *p* = 0.66; Extinction: males vs intact, *p* = 0.11; males vs OVX, *p* = 0.27; intact vs OVX, *p* = 0.002, Dunnett T3 corrected), but not during shock, where intact and OVX female rats experienced a similar reduction in foraging efficiency while male rats were largely able to maintain their baseline foraging efficiency (group: Shock: χ^2^_2_ = 7.66, *p* = 0.022, Extinction: χ^2^_2_ = 0.56, *p* = 0.76, Kruskal–Wallis tests; condition: *F*_(1.24,26.10)_ = 18.34, *p* = 0.000094, df adjusted; *post hoc* tests: Shock: males vs intact, *p* = 0.048; males vs OVX, *p* = 0.099; intact vs OVX, *p* = 1.00, Dunnett T3 corrected). While males tended to obtain meals more frequently (Fig. 3*A*; group: *F*_(2,21)_ = 4.20, *p* = 0.029; condition: *F*_(2,42)_ = 8.92, *p* = 0.00059; condition*group: *F*_(4,42)_ = 1.33, *p* = 0.28; *post hoc* tests: males vs intact, *p* = 0.25; males vs OVX, *p* = 0.028; intact vs OVX, *p* = 0.93, Bonferroni corrected), meal frequency decreased during the shock period similarly from baseline across all groups (Fig. 3*B*; group: *F*_(2,21)_ = 0.61, *p* = 0.55; condition: *F*_(2,42)_ = 8.39, *p* = 0.00086; condition*group: *F*_(2,42)_ = 1.04, *p* = 0.40). However, males specifically increased the mean size of each meal obtained during the shock period (Fig. 3*C*, *D*), whereas the meal sizes of intact and OVX females decreased during shock (raw data: group: *F*_(2,21)_ = 3.04, *p* = 0.070; condition: *F*_(2,42)_ = 14.94, *p* = 0.000013; condition*group: *F*_(4,42)_ = 4.95, *p* = 0.0023; *post hoc* tests: Baseline: males vs intact, *p* = 1.00; males vs OVX, *p* = 0.36; intact vs OVX, *p* = 0.087; Shock: males vs intact, *p* = 0.028; males vs OVX, *p* = 0.37; intact vs OVX, *p* = 0.67; Extinction: males vs intact, *p* = 1.00; males vs OVX, *p* = 0.34; intact vs OVX, *p* = 0.048; Bonferroni corrected*;* normalized data: group: *F*_(2,21)_ = 3.69, *p* = 0.042; condition: *F*_(2,42)_ = 12.59, *p* = 0.000052; condition*group: *F*_(4,42)_ = 4.06, *p* = 0.0072; *post hoc* tests: Shock: males vs intact, *p* = 0.018; males vs OVX, *p* = 0.006; intact vs OVX, *p* = 1.00; Extinction: males vs intact, *p* = 1.00; males vs OVX, *p* = 1.00; intact vs OVX, *p* = 1.00; Bonferroni corrected). After adjusting for baseline differences in body weight (Fig. 3*E*; group: *F*_(2,21)_ = 95.09, *p* = 2.98E-11; condition: *F*_(1.57,32.92)_ = 111.92, *p* = 3.06E-14, df adjusted; condition*group: *F*_(3.14,32.92)_ = 28.46, *p* = 2.03E-9, df adjusted), normalized growth rates (Fig. 3*F*) reveal that male rats are able to maintain a relatively stable growth trajectory compared to intact and OVX females (group: *F*_(2,21)_ = 4.69, *p* = 0.021; condition: *F*_(1.56,32.81)_ = 92.90, *p* = 4.27E-13, df adjusted; condition*group: *F*_3.13, 32.81_ = 15.75, *p* = 0.000001, df adjusted; *post hoc* tests: Baseline: males vs intact, *p* = 0.001; males vs OVX, *p* = 0.095; intact vs OVX, *p* = 0.19; Shock: males vs intact, *p* = 0.021; males vs OVX, *p* = 0.33; intact vs OVX, *p* = 60; Extinction: males vs intact, *p* = 0.001; males vs OVX, *p* = 0.006; intact vs OVX, *p* = 1.00; Bonferroni corrected).

**Figure 4.**
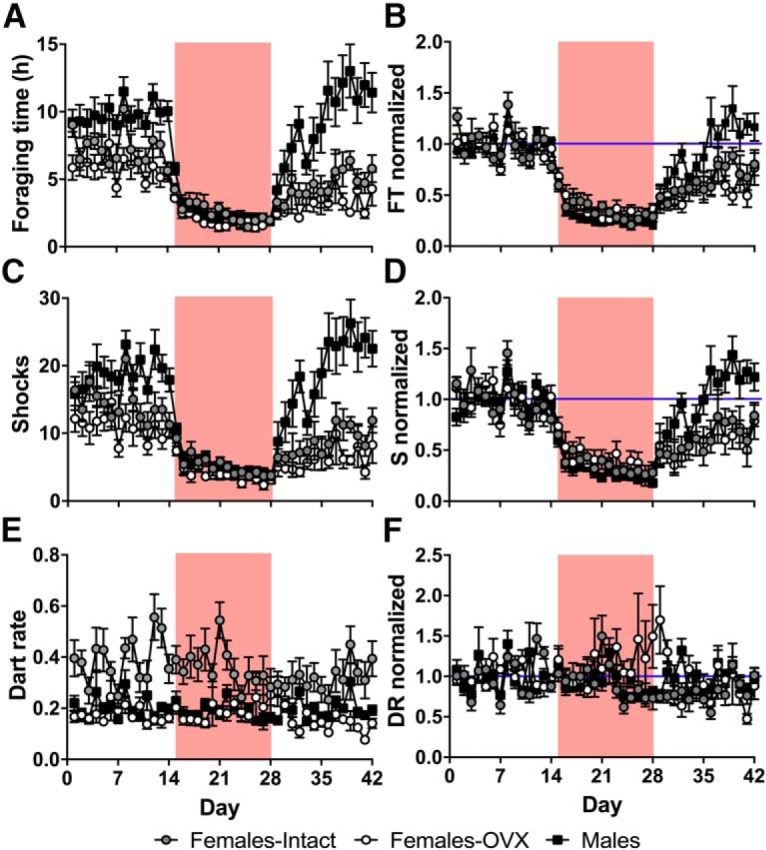
Sex differences and effects of ovariectomy and footshocks on defensive behaviors. ***A***, ***B***, Mean daily (***A***) and normalized (***B***; percentage of baseline average) time spent in the foraging area (percentage of total time; FT); ***C***, ***D***, mean daily (***C***) and normalized (***D***) expected (during baseline and extinction) and actual (during shock) number of shocks received (S). ***E***, ***F***, mean daily (***E***) and normalized (***F***) dart rate (in darts/min; DR). Gray circles represent intact female rats (*n* = 8), open circles represent OVX female rats (*n* = 8), and black squares represent male rats (*n* = 8). Red background denotes shock period. All data represent the mean ± SEM.

In terms of fear- and anxiety-related behaviors, although males spent more time foraging during baseline (Fig. 4*A*; group: *F*_(2,21)_ = 13.09, *p* = 0.00020; condition: *F*_(2,42)_ = 70.13, *p* = 4.11E-14; condition*group: *F*_(4,42)_ = 7.40, *p* = 0.00013; *post hoc* tests: Baseline: males vs intact, *p* = 0.080; males vs OVX, *p* = 0.0035; intact vs OVX, *p* = 0.55; Shock: males vs intact, *p* = 1.00; males vs OVX, *p* = 0.26; intact vs OVX, *p* = 0.29; Extinction: males vs intact, *p* = 0.0026; males vs OVX, *p* = 0.00019; intact vs OVX, *p* = 0.84; Bonferroni corrected), all groups exhibited similar reductions in the time spent in the foraging area following the introduction of footshocks (Fig. 4*B*; group: *F*_(2,21)_ = 1.75, *p* = 0.20; condition: *F*_(1.31,27.43)_ = 128.23, *p* = 6.38E-13, df adjusted; condition*group: *F*_(2.61,27.43)_ = 6.55, *p* = 0.0025, df adjusted; *post hoc* tests: Shock: males vs intact, *p* = 0.53; males vs OVX, *p* = 0.32; intact vs OVX, *p* = 1.00; Extinction: males vs intact, *p* = 0.067; males vs OVX, *p* = 0.024; intact vs OVX, *p* = 1.00; Bonferroni corrected). Figure 4*C* shows the expected number of shocks rats would have received based on their naive, baseline foraging behavior, the actual mean number of shocks each group received during the shock condition (triggered randomly and unsignaled, ∼2/h), and the expected mean number of shocks they would have received based on their foraging behavior during extinction (no shock), demonstrating that while males spent more time in the foraging area during baseline, all rats quickly learned to avoid footshocks and received a similar number of shocks during the shock period (group: *F*_(2,21)_ = 14.08, *p* = 0.00013; condition: *F*_(2,42)_ = 59.23, *p* = 5.96E-13; condition*group: *F*_(4,42)_ = 8.00, *p* = 0.000069; *post hoc* tests: Baseline: males vs intact, *p* = 0.14; males vs OVX, *p* = 0.0039; intact vs OVX: *p* = 0.37; Shock: males vs intact, *p* = 1.00; males vs OVX, *p* = 0.27; intact vs OVX, *p* = 0.26; Extinction: males vs intact, *p* = 0.0013; males vs OVX, *p* = 0.000099; intact vs OVX, *p* = 0.87; Bonferroni corrected). Furthermore, males extinguished this avoidance behavior at much faster rates than both intact and OVX female rats (Fig. 4*D*; group: *F*_(2,21)_ = 1.87, *p* = 0.18; condition: *F*_(1.44,30.23)_ = 96.38, *p* = 1.95E-12, df adjusted; condition*group: *F*_(2.88,30.23)_ = 7.26, *p* = 0.00095, df adjusted; *post hoc* tests: Shock: males vs intact, *p* = 1.00; males vs OVX, *p* = 0.37; intact vs OVX, *p* = 1.00; Extinction: males vs intact, *p* = 0.029; males vs OVX, *p* = 0.021; intact vs OVX, *p* = 1.00; Bonferroni corrected). Since it has recently been suggested that darting behavior—sudden activity bursts (exceeding 23.6 cm/s)—is a sexually dimorphic defensive behavior exhibited primarily by female rodents (Gruene et al., 2015), darting was examined throughout the present experiment in the same manner. While intact females displayed a greater number of darting episodes (Fig. 4*E*) than both males and OVX females during baseline (group: Baseline: χ^2^_2_ = 13.55, *p* = 0.0011; Shock: χ^2^_2_ = 10.99, *p* = 0.0041 Extinction: χ^2^_2_ = 10.60, *p* = 0.0050, Kruskal–Wallis test; condition: *F*_(2,42)_ = 4.32, *p* = 0.020; *post hoc* tests: males vs intact, *p* = 0.024; males vs OVX, *p* = 0.59; intact vs OVX, *p* = 0.011; Dunnett T3 corrected), there was an overall reduction in darting behavior during the shock and extinction conditions, and there were no sex-specific changes across experimental conditions in darting behavior after adjusting for baseline differences, indicating that darting may not be a specific defensive behavior (Fig. 4*F*) (group: *F*_(2,21)_ = 0.70, *p* = 0.51; condition: *F*_1.53, 32.15_ = 2.00, *p* = 0.16, df adjusted; condition*group: *F*_3.06, 32.15_ = 1.20, *p* = 0.33, df adjusted).

**Figure 5.**
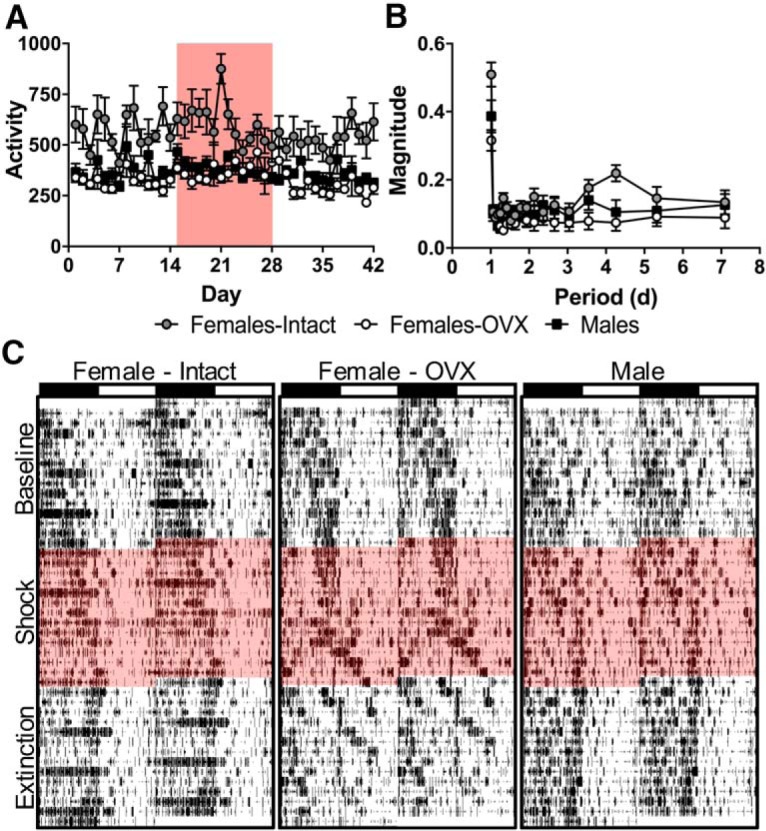
Sex differences and effects of ovariectomy and footshocks on general activity. ***A***, Mean daily activity (distance traveled, in meters). ***B***, Mean magnitude of component behavioral rhythms (converted to periods) derived via FFT of 1 h binned activity data (distance traveled, in meters) during baseline. Gray circles represent intact female rats (*n* = 8), open circles represent OVX female rats (*n* = 8), and black squares represent male rats (*n* = 8). All data represent the mean ± SEM. ***C***, Raster plots of activity (1 min bins) across all experiment days and conditions of representative intact female (left), OVX female (center), and male (right) rats. Red background denotes shock period.

Like darting, intact females demonstrated a greater level of general activity compared with both males and OVX females (Fig. 5*A*), which was relatively stable between groups and conditions (group: Baseline: χ^2^_2_ = 11.63, *p* = 0.0030; Shock: χ^2^_2_ = 11.45, *p* = 0.0033; Extinction: χ^2^_2_ = 12.89, *p* = 0.0016, Kruskal–Wallis test; condition: *F*_(2,42)_ = 7.67, *p* = 0.0014; *post hoc* tests: males vs intact, *p* = 0.018; males vs OVX, *p* = 0.63; intact vs OVX, *p* = 0.0074; Dunnett T3 corrected), although there were significant, albeit small, changes in overall activity across shock and extinction from baseline (group: *F*_(2,21)_ = 0.068, *p* = 0.93; condition: *F*_(2,42)_ = 7.51, *p* = 0.0016; condition*group: *F*_(4,42)_ = 0.35, *p* = 0.84). As other studies have found that the locomotor activity of females fluctuates with their estrous cycle (Birke and Archer, 1975; Blizard et al., 1975; Frye et al., 2000), locomotor activity in the closed economy was examined for rhythmic fluctuations. A Fourier analysis of the locomotor behavior of rats was performed, and the magnitudes of the component frequencies in the 1–7 d range were averaged by sex. As Figure 5*B* shows, all groups exhibited normal circadian rhythms (1 d periods), but intact females uniquely exhibited a peak in the 3–5 d range, which corresponds to the normal period range of the estrous cycle (4.26 d period: intact vs males: *t*_(14)_ = 2.66, *p* = 0.019; intact vs OVX: *t*_(14)_ = 4.24, *p =* 0.00083; males vs OVX: *t*_(14)_ = 0.72, *p* = 0.49). Raster plots of a representative female (Fig. 5*C*) exemplify this periodic increase in locomotion, which is not seen in representative male or OVX female animals.

**Figure 6.**
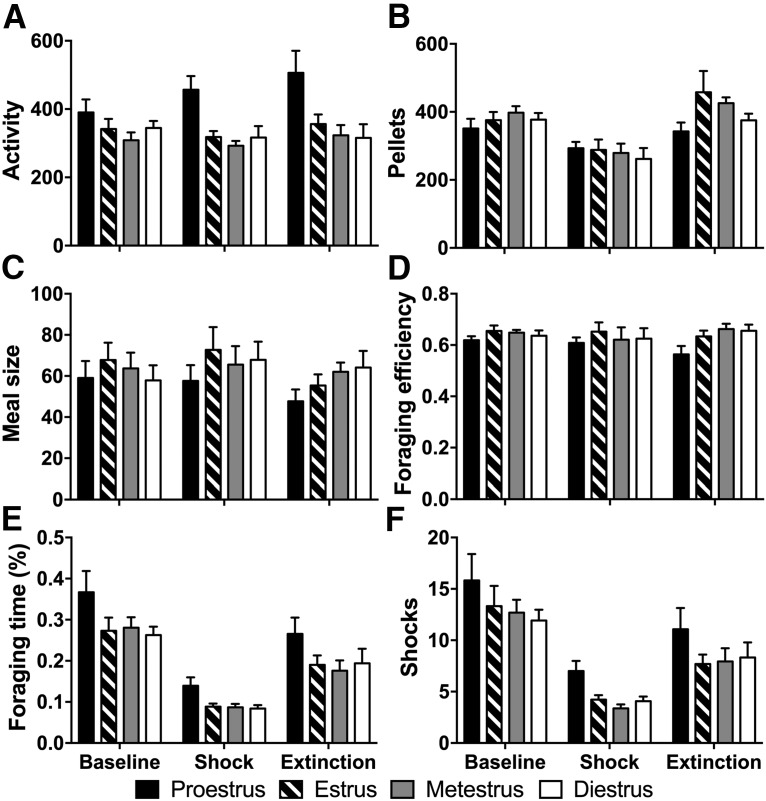
Effects of estrous phase on foraging and avoidance behaviors. ***A***, Activity (distance traveled, in meters). ***B***, Number of pellets obtained. ***C***, Meal size (pellets/meal). ***D***, Foraging efficiency (pellets/lever press). ***E***, Time spent foraging (percentage of total time). ***F***, Expected (during baseline and extinction) and actual number of shocks (during shock) received during proestrus (black), estrus (striped), metestrus (gray), and diestrus (white) phases averaged within each condition (baseline, shock, extinction).

To determine how estrous phase may influence foraging and defensive behaviors, estrous phase was determined daily in a subset of the intact females (*n* = 4) via vaginal lavage. Behaviors were averaged across individual rats for each phase of the estrous cycle within each experimental condition. As suggested by the FFTs, locomotor activity was significantly influenced by the phase of estrous, with increases in locomotion being associated primarily with the proestrus phase [Fig. 6*A*; *F*_(3,33)_ = 15.55, *p* < 0.001; Proestrus (P) vs Diestrus (D): *t*_(35)_ = 3.86, *p* < 0.001; P vs Estrus (E): *t*_(35)_ = 3.51, *p* = 0.001; P vs Metestrus (M): *t*_(35)_ = 3.23, *p* = 0.003]. While there were no phase effects on the amount of food consumed (Fig. 6*B*) or the sizes of meals obtained (Fig. 6*C*) throughout the experiment, proestrous phase was associated with a significant reduction in foraging efficiency (Fig. 6*D*; *F*_(3,33)_ = 3.35, *p* = 0.031; P vs D: *t*_(37)_ = 2.43, *p* = 0.020; P vs E: *t*_(37)_ = 3.12, *p* = 0.003; P vs M: *t*_(37)_ = 3.02, *p* = 0.005). Additionally, proestrus was associated with more time spent in the foraging zone (Fig. 6*E*; *F*_(3,33)_ = 7.21, *p* = 0.001; P vs D: *t*_(37)_ = 3.69, *p* = 0.001; P vs E: *t*_(37)_ = 3.23, *p* = 0.003; P vs M: *t*_(37)_ = 2.88, *p* = 0.007] and, subsequently, more shocks (Fig. 6*F*; *F*_(3,33)_ = 4.48, *p* = 0.010; P vs D: *t*_(36)_ = 3.32, *p* = 0.002; P vs E: *t*_(36)_ = 2.97, *p* = 0.005; P vs M: *t*_(36)_ = 2.62, *p* = 0.013). Thus, estrous phase does appear to influence risky foraging decisions, with increased risk taking associated with the proestrus phase, although this does not appear to be associated with an increase in gains (e.g., more food), but rather with a decrease in foraging efficiency.

## Discussion

The current study indicates that fear and anxiety function differently with respect to foraging behavior in male and female rats living in a dynamic and risky environment. The risky closed economy experiment demonstrated that, while both males and females avoid unpredictable shock at the same rate, they appeared to do so by using different foraging strategies. Whereas males responded to danger by increasing their meal size to maintain their baseline foraging efficiency and baseline body weight, females instead sacrificed their metabolic needs and lost weight rather than risk encountering the threat of footshock. Thus, the present findings extend the previous findings of sexual dimorphisms in fear and anxiety behavior, where male rats spent more time out of cover in a novel environment than female rats (Jolles et al., 2015), and female rats were more reluctant to consume food pellets in a novel environment than male rats (Carrier and Kabbaj, 2013; Turner and Burne, 2014), to risky foraging behavior embodying the larger-scale spatiotemporal integration of information that animals rely on to function in natural environments.

Differences between intact and ovariectomized females were primarily limited to overall body weight and the amount of food consumed. OVX females obtained more pellets per meal than intact females and were less active overall. Their baseline behaviors were more equivalent to male behavior, but they were similarly affected by the presence of shock and exhibited similar extinction rates to that of the intact females. Importantly, it has been shown that the estrous cycle influences pain sensitivity and that ovariectomized female rats exhibit higher pain thresholds than intact female rats (Ji et al., 2003, 2006; Prusator and Greenwood-Van Meerveld, 2016). In the present study, intact and OVX females exhibited similar rates of avoidance and changes in meal patterns during the shock period. If the pain threshold differed between intact and OVX groups, this did not appear to influence the long-term behavioral response to shock, which corresponds to others’ findings that lower pain thresholds do not necessarily translate to enhanced fear responses (Van Oyen et al., 1979; Kosten et al., 2006; Graham et al., 2009; Lehner et al., 2010; Schaap et al., 2013). The FFTs detected a significant difference in infradian range (3–5 d) periodicity between OVX and intact females, providing evidence that fluctuations in general activity are due to the estrous cycle. While the proestrus phase of the estrous cycle in intact females was associated with an increase in risky behavior and more shocks received during the shock period, this was met with a decrease in foraging efficiency. Thus, the function of proestrus-related modulation of risky behavior does not appear to be directed toward facilitating foraging needs, and instead may be more related to reproductive behaviors, as previously suggested (Archer, 1975; Morgan et al., 2004; Byrnes and Bridges, 2006). Although it is clear that ovarian hormones play a relatively small activational role in the day-to-day behavior of female rats, they may not need to be actively controlled in experimental studies of fear and anxiety if estrous phase-specific phenomenon are not of interest, as others have suggested (Prendergast et al., 2014), and may be measured indirectly in locomotor behavior in studies that use similar longitudinal measurement. It is well established that ovarian hormones are critical during early development (Beatty, 1979; van Haaren et al., 1990), and such organizational effects and their functional consequences for fear, anxiety, and foraging behavior should be examined in future studies.

The risky closed economy design is akin to a continual inhibitory avoidance situation wherein footshock is a diffuse, unpredictable threat associated with a specific place (the gridded foraging area), and avoidance of shock is dependent on the inhibition of movement into the dangerous area. The finding that females extinguish slower than males in the closed economy is contrary to previous findings in single-footshock inhibitory avoidance paradigms, which show that females are quicker to return to an area associated with shock than males (Van Oyen et al., 1979; Heinsbroek et al., 1988; Beatty et al., 2013). The simultaneous approach food–avoid footshock conflict and relatively uninterrupted, long-term behavioral assay in this experiment may explain this discrepancy, which suggests that traditional inhibitory avoidance paradigms might not adequately address important functional and temporal dynamics of fear and anxiety as they relate to natural behaviors. Furthermore, the sex differences in general activity and darting behaviors during baseline and their corresponding reductions during the shock period cast doubt on the validity of tests that depend largely on locomotion (or its absence, as in freezing) for examining fear and anxiety behavior, at least when the threat is relatively diffuse, such as in open field and elevated-plus maze tests. These studies have typically concluded that female rodents are less fearful than male rodents as females are more likely to enter the center of an open space or the open arms of the elevated plus maze (both interpreted as risky behaviors; Masur, 1972; Blizard et al., 1975; Johnston and File, 1991; Zimmerberg and Farley, 1993). As others have argued (Masur, 1972; Gray and Lalljee, 1974; Archer, 1975; Birke and Archer, 1975; Fernandes et al., 1999), greater locomotor activity in females appears to be more related to general metabolic factors, which ovarian hormones are known to influence (Campbell and Febbraio, 2001), rather than fear or anxiety. Finally, the current results provide a closer match to sex differences in fear and anxiety observed in humans compared with Pavlovian fear-conditioning paradigms. Thus, approach–avoidance conflict situations may be more relevant to translational approaches to studying pathological fear and anxiety.

What, then, is the basis of sex differences in risky foraging strategies? The fact that ovariectomy in adult female rats did not alter the risky foraging strategies they used suggests that such behaviors are prepared during development. Evolutionary–developmental theory suggests that male and female mammals can be expected to display divergent behaviors as they typically make different trade-offs regarding the maintenance of survival and reproduction (Kodric-Brown and Brown, 1987; Ellis et al., 2009; West-Eberhard, 2014). Females tend to allocate more bioenergetic effort toward offspring development (i.e., parenting), while males tend to allocate more effort toward reproducing, and the propensity of a male to take risks may facilitate a reproductively focused strategy by promoting the achievement of social dominance and gaining access to mates (Kodric-Brown and Brown, 1987; Steinberg, 2008; Jolles et al., 2015). That females exhibit an attenuation in fear and anxiety behaviors just before the estrus phase may be related to their evolutionary need to mate despite the potential violence commonly accompanying mating behavior in many animals (West-Eberhard, 2014). Indeed, the presence of males, or just their odor, has been shown to elicit a stress response in rats (Sorge et al., 2014; Shors et al., 2016). Thus, proestrus-related hormones may function to decrease female aversion to copulation, and its effects on fear learning in laboratory settings may be an artifact of such a function. Following this interpretation, it is reasonable that a motivational stimulus relating to reproduction, such as access to a mate, may reveal a different pattern of estrous-related changes in risky decision-making.

In summary, the closed economy approach used in this study allows for the simulation of threat contingencies that animals are likely to encounter in the wild. In contrast to fear-conditioning studies that have reported that male rats show greater conditioned fear responses than female rats, the present results indicate that when animals live for extended periods, largely undisturbed, in a semi-naturalistic environment, female rats are more reactive to environmental threats than male rats, which models anxiety and fear-related disorders in humans. Future studies may benefit from similar longitudinal approach–avoidance paradigms and diverse measures to explore threat situations and biological factors (e.g., putative fear circuitry) underlying sex differences in fear behavior.

## Synthesis

Reviewing Editor: Carmen Sandi, Swiss Federal Institute of Technology Decisions are customarily a result of the Reviewing Editor and the peer reviewers coming together and discussing their recommendations until a consensus is reached. When revisions are invited, a fact-based synthesis statement explaining their decision and outlining what is needed to prepare a revision will be listed below.

The reviewers and review editor consider this work highly interesting and well performed. It provides a novel neurobehavioral model in rats and, importantly, considers sex-related differences. The results are sound and important. We would like to ask the authors to incorporate the different points raised by the reviewers (see list with specific points below) in the corresponding parts of the text; as you will see from the reviewers comments included below, this would require including different wording, new references or literature re-framing, new analyses, or commentaries as limitations or additional explanations. Note that we have agreed that the Major points raised by reviewer 1 do not need to be addressed by performing new experiments; instead, they should be carefully addressed as modifications in the text, as either points to take into account in the discussion (points 1 and 2) or new analyses of existing data (point 3).

Reviewer 1:

The present study set out to investigate how rats of both sexes behave in a situation where animals have competing drives modulating their approach to the same environment: the motivation to obtain food and the need to avoid electrical shocks. This is a paradigm that allows the execution of richer and maybe more natural rat behaviors over longer periods of time.

This idea is not new. It has been previously developed by others (see Helmstetter and Fanselow 1993 which the authors reference), but in this study females were also included (intact and ovariectomized females), which has been very rare in research in general. Females are usually only used when the subject of study is directly related to female reproductive behavior. However, for a variety of reasons, it is finally acknowledged that females must be included in basic and translational studies: there are sexual dimorphism between the two sexes and females “suffer the nuisance” of the menstrual cycle that in many cases leads to increased variability in the results. However, that variability, more than a bug, it reflects the different biology of the two sexes and by itself must be acknowledged and studied. Thus, this study is novel since it included males, intact females and ovariectomized females.

The authors found that both genders avoid unsignaled shocks at the same rate, but they do it differently. While male rats increase their meal size and maintain their pressing efficiency, managing to maintain their growth rate despite a decrease in the number of pellets eaten, females decreased markedly their food intake at the expense of their growth (both their level pressing and efficiency go down). The reproductive cycle had some effects on the baseline performance of females (very interesting, OVX females eat more than intact females and this is achieved in part by high levels of efficiency in lever pressing, even higher than males) but during shock, the foraging efficiency of OVX females drops to the levels of intact females, pointing to a dimorphism that is set through development and doesn't depend on the activational effect of sex hormones during adulthood.

I like this study very much and there are only a couple major points that I would like to be addressed.

Major points:

1. Pain thresholds: the same shocks were used for all groups of animals. Do we actually know if females (OVX and intact) and males feel the shocks the same way? It would be helpful to have an independent test to assess pain threshold and make sure that the behavioral differences in the response to shocks are not due to this. Also, would it be possible that pain thresholds change during the reproductive cycle. Did the authors assess this? Is it possible to do it?

2. I would like the authors to put their work in the context of Helmstetter and Fanselow, 1993, in particular the dimorphism that they observe in terms of mitigation. In Helmstetter and Fanselow, 1993, rats were exposed to different numbers of shocks per day (12, 24, 48 or 96 shocks per day). 48 shocks per day (which is what is used in this ms: 2 shocks per hour, 48 shocks per day) gave very similar results between the two studies, which is very nice to observe. However, other shock frequencies gave very different results, in particular a higher shock rate led to results in the male that are very similar to the ones observed in females in the current study. So, I wonder if the dimorphism observed in foraging strategy during shocks can be explained by the intensity of the shocks or the rate. Is this a sweet spot (48 shocks per day at this intensity) where sexual dimorphism is exhibited? Or is this difference maintained over different shock rates/intensities?

3. Other behaviors? The authors have access to many more behavioral readouts that are not included in the ms and that could make the story more interesting and which are valuable for the community in general. Freezing behavior? Do males and females freeze the same manner when they go to the nest? Latencies to return to the foraging area after shock? For example, males increase their meal size during shock: do they have any strategy to perform that? Shocks are randomly distributed, but I guess the probability of two shocks being delivered very close in time is low. Do males tend to increase the meal size after a shock is delivered? Is there any difference in that behavior between females in different states of the cycle? Do animals dig in the nest? It would be nice if the authors had other behaviors that could directly measure fear so that they could really say that they have a paradigm to study it, rather than saying it indirectly by the avoidance behavior?

Minor points:

- In the last figure the authors show the data for female rats by estrous state. I guess the authors argue that there is no activational hormonal effect that can explain the differences between males and females because the same pattern for the behaviors that are studied across the cycle is maintained across the three conditions (baseline, shock and extinction) and because OVX and intact females behave very similar during shock condition. However, it would be nice to see the comparison between OVX females and the 4 different states of the intact females. Are OVX females more similar to a proestrous female or a diestrous?

- This is not my field, but I would be more careful in the wording of the abstract:

Line40: I am not from the field, but the authors do not measure directly any fear or anxiety related behaviors; in my perspective they infer that by measuring changes in the foraging behavior.

“...to examine sex differences in fear- and anxiety-related behaviors” I would switch to “...to examine sex differences in foraging behavior”

Line46/47: from what I understood, OVX females behave more like males during baseline and extinction periods, but during the shock phase they behave like intact females. Therefore, I do not think it is correct to say that “ovarian hormone fluctuations were shown to exert slight but reliable rhythmic effects on risky decision-making”, rather it should be “ovarian hormone fluctuations were shown to exert slight but reliable rhythmic effects on foraging strategies”. Actually, the rest of the sentence is in disagreement with this statement.

- It is interesting that the reproductive cycle does not seem to have an influence in the strategy that females adopt during shock condition, since they behave very similar to OVX females. However, it would be interesting to see how these females would behave in a context where the competition was done between shock and a sexually relevant stimulus. It is known that the estrous state modulates metabolic needs (which does not seem to influence in this case female behavior), but it would be nice if the authors at least included this point in their discussion. I do not think it is fair to say that we do not need to worry about the estrous state in this type of risky task, it may be that the estrous cycle might influence female behavior differently depending on the stimulus identity.

Reviewer 2:

The present study used a “closed economy” system to assess risky and anxiety-related behaviors. In this system, rats had to risk getting a footshock in order to gain access to food and water. This conflict caused a sexual divergence in behavior, such that males foraged less but ate larger meals when they did venture out, whereas females foraged less and consumed less food and water. These behavioral differences are interesting, but I have some questions/comments re. the interpretation of these results and other design elements that are detailed below.

1) The authors describe that estrous cycle-induced variability is an obstacle that people raise to including female rodents in studies. However, the authors need to clarify that this is a perceived obstacle, not a real obstacle. While the estrous cycle can, in some cases, account for variability in females, females are not more inherently more variable than males. Please refer to Prendergast, Onishi, and Zucker (2014) and Becker, Prendergast, and Liang (2016).

2) In terms of the significance statement, I do not agree that longitudinal designs mitigate the need to assess cycle or that a 42 day-long task is any easier that tracking cycle for a short task.

3) In the introduction, the point that males show more fear than females is only true if “freezing” is the measure of fear. Recent work by Gruene et al. (2015)-cited within this manuscript-demonstrate that when darting is used as an additional indices of fear, this male bias is mitigated.

4) Number of subjects where estrous cycle was tracked is very small (n=4). Why wasn't the cycle tracked in all intact females? Can the authors provide a power analysis to justify such a small “n”?

5) In the discussion, the authors note differences between the results of the present study and that of inhibitory avoidance procedures and then conclude that the discrepancy “suggests that traditional inhibitory avoidance paradigms might not adequately address important temporal dynamics of fear and anxiety behavior.” This is overstated. As the authors note, their task is a conflict task where food is only available on the side of the apparatus where shock is administered. This is very different than inhibitory avoidance and these differences are likely driving the discrepant finding. Discrepant findings do not suggest a well-validated model, like inhibitory avoidance is inadequate.

6) The challenge with studying sex differences in approach-avoidance tasks is that it is difficult to determine whether the sex difference is being driven by sex differences in avoidance (the authors conclusion) or approach. Compared to female rats, males are much bigger, consume much more food, and continue to grow through early adulthood (the period during which these rats are being tested). Couldn't the results simply reflect the fact that males are more motivated to consume pellets than females? The authors should address this issues.

7) Other approach avoidance tasks exist, such as novelty suppressed feeding and novelty induced hypophagia. Is there any literature suggesting that females are more avoidant in these tasks which could strengthen the authors' conclusions?
